# NASO-RVG (NR) Technique as a Novel Approach to Nasal Bone Radiography Using Radiovisiography and Portable X-ray: A Pilot Study

**DOI:** 10.7759/cureus.89405

**Published:** 2025-08-05

**Authors:** Prabhusankar K, Arundheepan P, Vigneswaran T, Priyadharsana PS, Effie Edsor, Vigneshwar S, Vignesh K

**Affiliations:** 1 Department of Oral and Maxillofacial Surgery, RVS Dental College and Hospital, Coimbatore, IND

**Keywords:** facial trauma imaging, nasal bone fracture, nasal radiography, naso-rvg technique, oral and maxillofacial surgery and facial trauma surgery, portable x-ray, radiovisiography (rvg), rvg

## Abstract

Introduction

Accurate imaging of nasal bone fractures is essential for proper diagnosis and management. Traditional methods such as lateral cephalograms and standard radiographs often suffer from limitations in resolution and positioning accuracy. This study introduces and evaluates a novel radiographic technique, that is, NASO-RVG (NR), utilizing radiovisiography (RVG) in combination with a portable X-ray unit for the improved visualization of nasal bone structures.

Objective

This study aimed to assess the efficacy and diagnostic clarity of the NR technique in nasal bone imaging when compared with conventional lateral cephalometric radiography.

Methods

A pilot study was conducted on 20 patients presenting with nasal bone fractures. Group A (n=10) underwent radiographic assessment using the NR technique, while Group B (n=10) received standard lateral cephalogram imaging. Parameters evaluated included image clarity, anatomical detail, ease of positioning, and diagnostic confidence, rated by blinded radiologists using a standardized scoring system.

Results

The NR technique demonstrated significantly improved visualization of fracture lines, better delineation of nasal bone contours, and higher diagnostic confidence scores (p<0.05) compared to the conventional lateral cephalogram. Additionally, patient positioning was easier and more reproducible with the NR technique.

Conclusion

The NR technique offers a reliable, high-resolution, and portable alternative for nasal bone imaging, with potential benefits in both emergency and outpatient settings. Further large-scale studies are recommended to validate these findings and establish clinical protocols.

## Introduction

Nasal bone fractures are the most frequently encountered facial skeletal injuries, accounting for nearly 40% of all facial bone fractures due to the prominent and exposed position of the nasal complex on the face [1]. Accurate imaging plays a vital role in diagnosis and treatment planning, given the aesthetic and functional consequences of nasal trauma. Conventional radiographic modalities such as lateral nasal radiographs, Waters view, and lateral cephalograms are commonly used to evaluate these fractures; however, each has limitations, including poor soft tissue resolution, anatomical superimposition, and inconsistent patient positioning [2].

Radiovisiography (RVG) is a digital imaging modality primarily utilized in dental practice, valued for its high image resolution, reduced radiation exposure, rapid image acquisition, and digital storage capabilities [3]. Despite its widespread use in dentistry, RVG has not been thoroughly explored for craniofacial imaging applications, such as the assessment of nasal bone injuries.

The use of portable X-ray systems has expanded significantly in trauma care, emergency departments, and low-resource environments where access to fixed radiographic units is often limited. When combined with RVG sensors, portable systems can facilitate high-quality skeletal imaging at the bedside or outpatient clinics, eliminating the logistical challenges of transporting trauma patients [4].

To address the limitations of conventional imaging, we developed the NASO-RVG (NR) technique, a novel approach that integrates RVG with a portable X-ray unit to obtain clear, high-resolution images of the nasal bones. This technique aims to overcome existing challenges by providing improved anatomical detail, enhanced image clarity, better positioning reproducibility, and diagnostic confidence, especially in settings where traditional radiographic equipment is inaccessible or impractical.

Therefore, this pilot study was conducted to compare the NR technique with conventional lateral cephalograms in terms of diagnostic clarity, image resolution, anatomical visibility, and reproducibility of patient positioning for the radiographic assessment of nasal bone fractures.

## Materials and methods

Study design and setting

This prospective, comparative pilot study was conducted in the Department of Oral and Maxillofacial Surgery at RVS Dental College and Hospital in Coimbatore, India, over a period of three months, from March 2025 to May 2025. The study was approved by the Institutional Ethical Committee of RVS Dental College and Hospital (approval number: 71/ETHICS/2024). Written informed consent was obtained from all patients prior to their inclusion in the study.

Study population

The study population consisted of 20 patients, aged between 18 and 50 years, who presented with clinical features suggestive of isolated nasal bone fractures. Patients with comminuted fractures, associated facial fractures, prior history of nasal surgery, or systemic bone disorders (such as osteoporosis or metabolic bone diseases) or those who exhibited uncooperative behavior were excluded from participation to ensure the homogeneity and reliability of radiographic evaluation.

Study groups

Eligible participants were randomly allocated into two groups of equal size. Group A (n=10) underwent radiographic evaluation using the NR technique, a novel method combining RVG with a portable X-ray device. Group B (n=10) underwent standard radiographic assessment using the conventional lateral cephalogram, serving as the control group.

Imaging protocols

**Figure 1:**
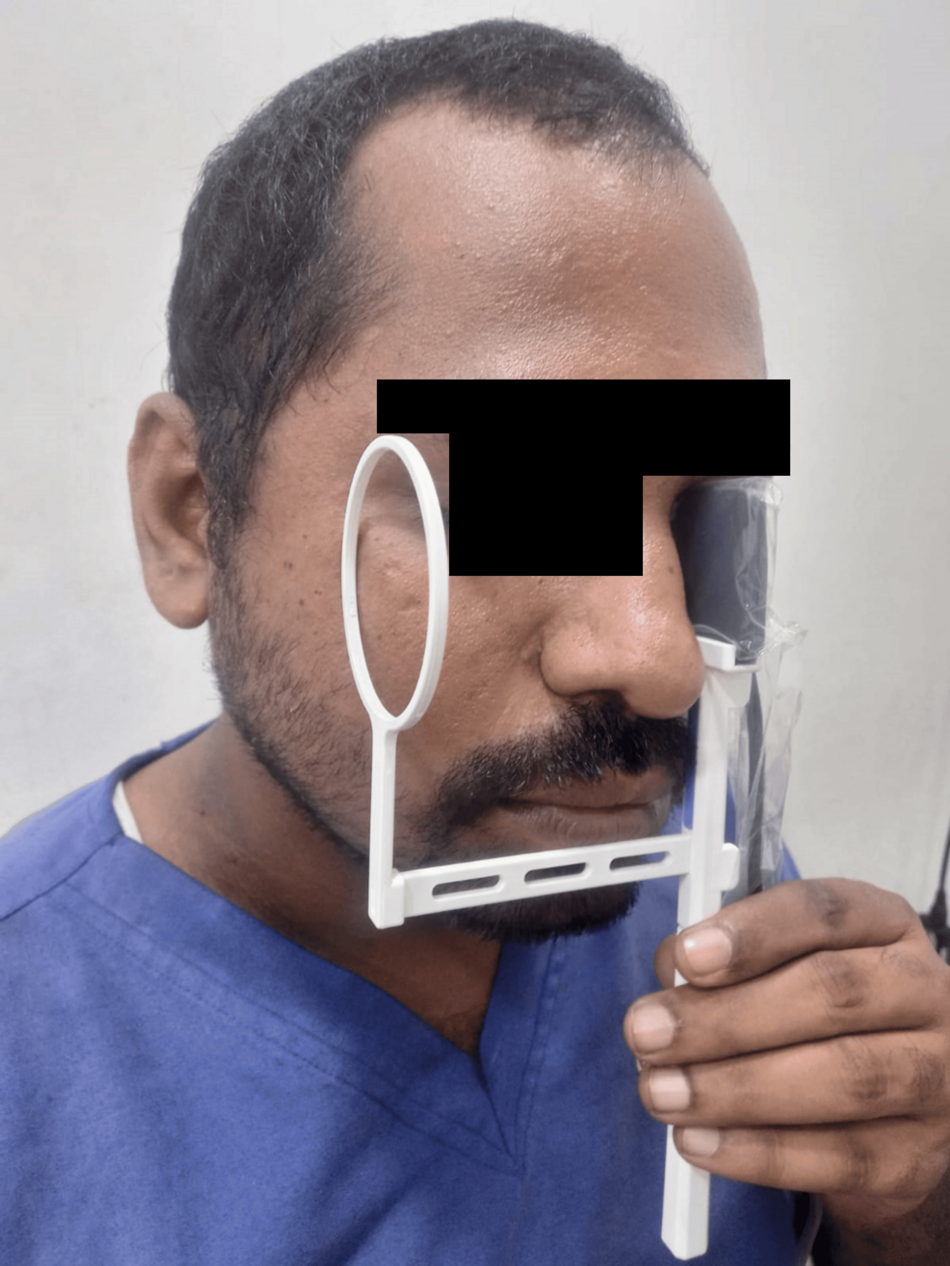
RVG sensor with the holder in the position for nasal bone radiography The figure shows the RVG sensor mounted on a custom-designed external holder and positioned over the nasal bridge. This standardized setup ensures optimal and reproducible alignment of the sensor for capturing clear radiographic images of the nasal bone anatomy during intraoperative or clinical assessment. RVG: radiovisiography

In the NR group, imaging was performed using a portable handheld X-ray unit (Runyes, Unicorn DenMart, Ningbo, China) combined with a digital RVG sensor (Xpect Vision, Unicorn DenMart, Ningbo, China). The patient was seated upright with the head stabilized for optimal positioning. A custom-designed sensor holder was used to externally mount the RVG sensor over the nasal bridge, allowing for standardized alignment of the X-ray cone perpendicular to the nasal region, as illustrated in Figure 1. This positioning facilitated consistent and high-quality radiographic images of the nasal bones across cases. Exposure settings were maintained at 65-70 kVp, 8 mA, and 0.32 seconds, and image capture was performed using the RVG imaging software.

For the lateral cephalogram group, conventional lateral cephalometric radiographs were obtained using a standard cephalostat unit. Patients were positioned following the standard cephalometric protocol to ensure consistent head orientation and image quality. The exposure settings were adjusted to 70 kVp, 10 mA, and 1.0 seconds. These settings allowed for the comprehensive visualization of the facial structures, including the nasal bones.

**Figure 2:**
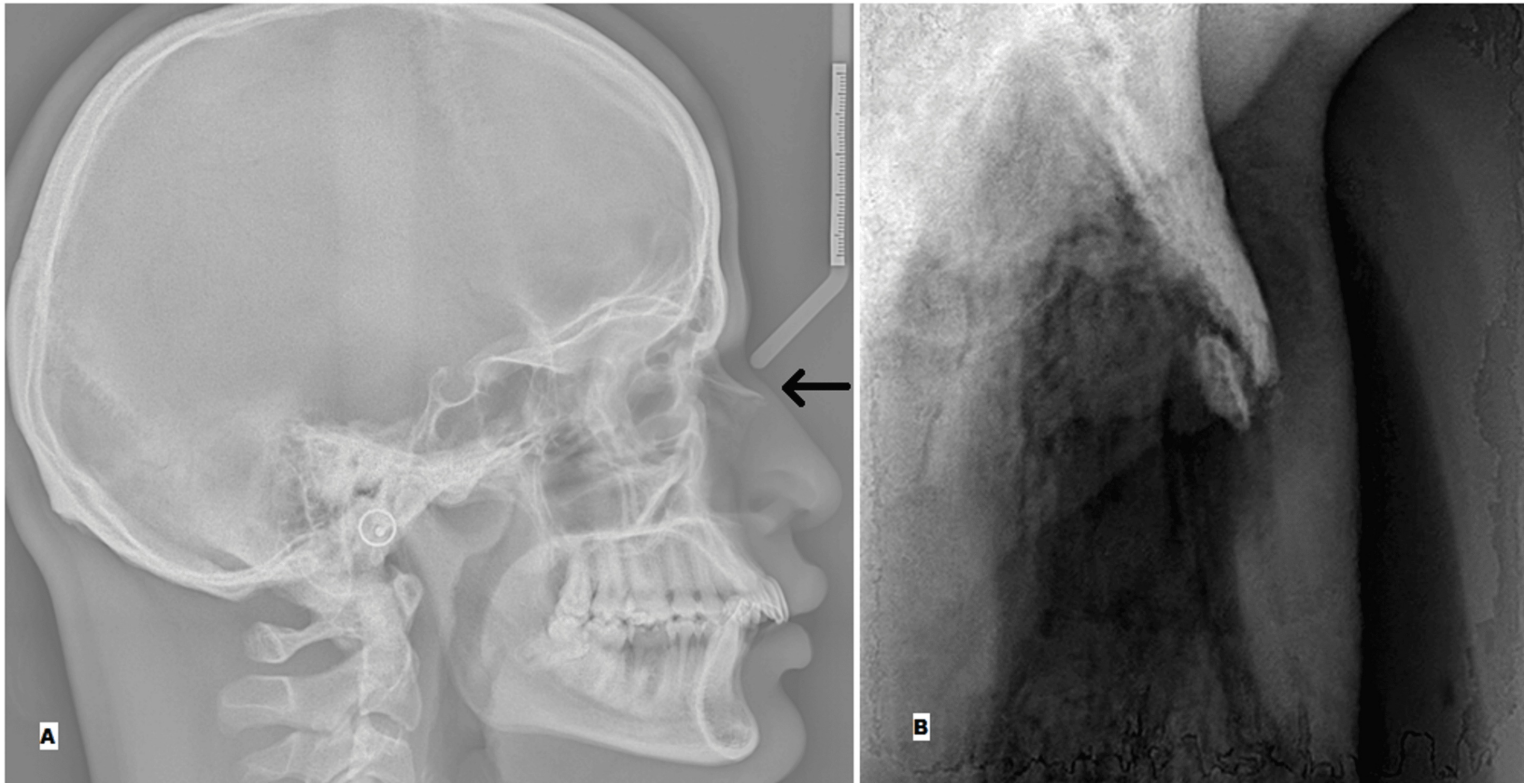
Comparison of lateral cephalogram and RVG in a patient with nasal bone fracture (A) Lateral cephalogram showing the craniofacial skeleton in sagittal view, with limited detail of the nasal bone and fracture line due to overlapping anatomical structures. The area of interest, that is, nasal bone region, is indicated by an arrow. (B) RVG image of the same patient focusing on the nasal bone region, demonstrating sharper visualization of the fracture line and bony contour due to improved resolution and sensor proximity. This side-by-side comparison highlights the enhanced diagnostic clarity of RVG imaging for nasal bone assessment, particularly in intraoperative and postoperative contexts. RVG: radiovisiography

For comparative evaluation, radiographic images from both modalities were analyzed in the same patient diagnosed with an isolated nasal bone fracture. The lateral cephalogram offered a broad sagittal view of the craniofacial skeleton, while the RVG provided a closer, more focused image of the nasal region. Notably, the RVG revealed a clearer fracture line and enhanced bone margin definition due to its higher resolution and sensor proximity. The contrast between the two imaging modalities is illustrated in Figure 2, where each panel demonstrates their respective diagnostic capabilities.

Evaluation criteria

All radiographic images were independently evaluated by two experienced oral radiologists who were blinded to the imaging technique used for each case. The evaluation focused on five key parameters: image clarity and sharpness, visibility of the fracture line, reproducibility of patient positioning, anatomical detail including the nasal bone outline and articulations, and overall diagnostic confidence. Diagnostic confidence was assessed using a custom 5-point Likert scale, where 1 indicated very low confidence and 5 indicated very high confidence in diagnosing nasal bone fractures. This scale, although not externally validated, reflects standard clinical radiographic assessment practices. Each parameter was scored for every patient in both groups, and the scores were systematically tabulated.

In the event of a discrepancy greater than 1 point between the two evaluators for any parameter, a consensus score was obtained through joint re-evaluation and discussion. This ensured objectivity and consistency in the interpretation of imaging results.

Scoring and statistical analysis

The individual scores assigned by the two radiologists were recorded and compared across both groups. Cases requiring consensus were jointly reviewed to finalize the score. All data were compiled and analyzed using IBM SPSS Statistics for Windows, Version 20.0 (IBM Corp., Armonk, New York, United States). Descriptive statistics were employed to summarize the demographic characteristics of the study participants. Intergroup comparisons of ordinal data were conducted using the Mann-Whitney U test, while categorical variables were analyzed using the chi-squared test. A p-value of less than 0.05 was considered to indicate statistical significance.

## Results

A total of 20 patients were included in this study, with 10 patients in each group. The mean age of participants in Group A (NR) was 32.4±6.2 years, and in Group B (lateral cephalogram), it was 31.7±5.9 years. There was no statistically significant difference in age or gender distribution between the groups (p>0.05) (Table 1).

**Table 1:** Patient demographics

Parameter	Group A (NASO-RVG)	Group B (lateral cephalogram)	P-value
Number of patients	10	10	-
Mean age (years)	32.4±6.2	31.7±5.9	0.75
Male-to-female ratio	7:3	6:4	0.65

Radiographic evaluation revealed that the NR technique demonstrated superior performance across all parameters compared to the conventional lateral cephalogram (Table 2). Group A achieved significantly higher mean scores for image clarity and sharpness (4.7±0.3 vs. 3.6±0.5; p<0.01), visibility of fracture line (4.6±0.4 vs. 3.4±0.6; p<0.01), anatomical detail (4.8±0.2 vs. 3.5±0.5; p<0.01), and diagnostic confidence (4.6±0.3 vs. 3.4±0.5; p<0.01).

**Table 2:** Radiographic evaluation score

Parameter	Group A (mean±SD)	Group B (mean±SD)	P-value
Image clarity and sharpness	4.7±0.3	3.6±0.5	<0.01
Visibility of fracture line	4.6±0.4	3.4±0.6	<0.01
Anatomical detail	4.8±0.2	3.5±0.5	<0.01
Diagnostic confidence score	4.6±0.3	3.4±0.5	<0.01
Positioning reproducibility	4.5±0.3	3.3±0.6	<0.01

In addition, the reproducibility of patient positioning was also significantly better in the NR group (4.5±0.3) compared to the lateral cephalogram group (3.3±0.6) (p<0.01). These differences were statistically significant and consistent across both evaluators.

No complications or image acquisition failures were reported in either group. The use of the RVG holder/positioner ensured consistent sensor placement, contributing to better reproducibility and image quality in Group A.

## Discussion

Accurate radiographic evaluation of nasal bone fractures is essential for diagnosis and treatment planning, particularly when clinical assessment is compromised by swelling or subtle displacement. This pilot study explored the NR technique, a novel approach that integrates RVG with a portable X-ray unit. Our results suggest that this method may offer certain advantages over conventional lateral cephalograms, including potential for enhanced image clarity, improved patient positioning reproducibility, and subjective diagnostic confidence.

The superior image clarity observed with the NR technique is likely attributed to the high spatial resolution of RVG sensors, which are widely used in dental radiology due to their ability to capture fine anatomical details [5]. By adapting these sensors for nasal bone imaging using a standardized sensor holder and a portable X-ray unit, consistent and detailed visualization of fracture lines and nasal contours was achieved. This addresses some of the limitations of lateral cephalometric radiographs, such as anatomical superimposition and suboptimal soft tissue contrast [6].

An additional strength of the NR technique is the reproducibility of patient positioning. The compact nature of the equipment allowed for better control of both sensor and cone placement, reducing variability in image acquisition. This is particularly advantageous in emergency settings or outpatient environments where patient cooperation may be limited and time constraints are common [7]. The ability to instantly assess image quality and repeat the procedure when necessary reduces the likelihood of diagnostic errors and minimizes overall radiation exposure to patients [8].

Radiologists involved in image evaluation reported a higher level of diagnostic confidence when reviewing NR images compared to lateral cephalograms. However, it must be noted that diagnostic confidence was assessed using a 5-point Likert scale designed specifically for this study and not externally validated. This limits the objectivity and generalizability of this metric. Future studies should consider employing or developing validated tools to more rigorously assess diagnostic certainty.

While advanced imaging modalities such as computed tomography (CT) and cone-beam computed tomography (CBCT) remain the gold standard for complex facial fractures, their routine use in isolated nasal bone injuries may be limited by cost, radiation exposure, and availability, especially in rural or resource-constrained settings [9]. The NR technique offers a more accessible and lower-radiation alternative for such environments.

Despite these promising results, several limitations must be acknowledged. The small sample size restricts statistical generalizability. Interobserver agreement was not statistically quantified using tools such as kappa analysis. Furthermore, comparison with CT or CBCT imaging was not performed, and no objective image quality parameters (e.g., signal-to-noise ratio or edge sharpness) were measured. These factors limit the robustness of conclusions regarding diagnostic superiority.

Nonetheless, the NR technique demonstrates potential as a portable, cost-effective, and low-radiation imaging alternative, particularly suited to outpatient departments and healthcare settings lacking access to advanced imaging. As a preliminary investigation, this pilot study establishes a foundation for future research. Further multicenter studies with larger sample sizes, inclusion of validated diagnostic assessment tools, and objective image analysis metrics are needed to substantiate and expand on these findings.

## Conclusions

The NR technique shows promise as a potentially effective, portable, and high-resolution imaging modality for the evaluation of nasal bone fractures. In this preliminary study, it demonstrated improved image clarity, anatomical detail, and diagnostic confidence compared to conventional lateral cephalograms. The use of a standardized RVG holder/positioner also contributed to reproducible patient positioning. However, due to the limited sample size, these findings should be interpreted with caution, and further studies with larger, statistically powered cohorts are warranted to validate these results.

Given its simplicity, cost-effectiveness, and ease of implementation in outpatient and emergency settings, the NR technique may serve as a practical alternative for nasal bone radiography, especially in resource-limited environments. Further large-scale studies and comparisons with advanced imaging modalities like CT or CBCT are warranted to validate these findings and support broader clinical adoption.
